# Advanced LC–MS-based methods to study the co-occurrence and metabolization of multiple mycotoxins in cereals and cereal-based food

**DOI:** 10.1007/s00216-017-0750-7

**Published:** 2017-12-22

**Authors:** Alexandra Malachová, Milena Stránská, Marta Václavíková, Christopher T. Elliott, Connor Black, Julie Meneely, Jana Hajšlová, Chibundu N. Ezekiel, Rainer Schuhmacher, Rudolf Krska

**Affiliations:** 1 Center for Analytical Chemistry, Department of Agrobiotechnology (IFA-Tulln), University of Natural Resources and Life Sciences, Vienna (BOKU), Konrad Lorenz Str. 20, 3430 Tulln, Austria; 2 Department of Food Analysis & Nutrition, Faculty of Food & Biochemical Technology, University of Chemistry & Technology, Technická 3, 166 28 Prague 6, Czech Republic; 3 Institute for Global Food Security, School of Biological Sciences, Queens University Belfast, 18-30 Malone Road, Belfast, BT9 5BN UK; 4 Department of Microbiology, Babcock University, Ilishan Remo, Ogun State 121103 Nigeria

**Keywords:** Fungal secondary metabolites, Liquid chromatography–tandem mass spectrometry, Liquid chromatography–high-resolution mass spectrometry, Metabolomics, Validation

## Abstract

Liquid chromatography (LC) coupled with mass spectrometry (MS) is widely used for the determination of mycotoxins in cereals and cereal-based products. In addition to the regulated mycotoxins, for which official control is required, LC–MS is often used for the screening of a large range of mycotoxins and/or for the identification and characterization of novel metabolites. This review provides insight into the LC–MS methods used for the determination of co-occurring mycotoxins with special emphasis on multiple-analyte applications. The first part of the review is focused on targeted LC–MS approaches using cleanup methods such as solid-phase extraction and immunoaffinity chromatography, as well as on methods based on minimum cleanup (quick, easy, cheap, effective, rugged, and safe; QuEChERS) and dilute and shoot. The second part of the review deals with the untargeted determination of mycotoxins by LC coupled with high-resolution MS, which includes also metabolomics techniques to study the fate of mycotoxins in plants.

**Electronic supplementary material:**

The online version of this article (10.1007/s00216-017-0750-7) contains supplementary material, which is available to authorized users.

## Introduction

Cereals and cereal-based products are the most important commodities in human nutrition. The health benefits of whole grain cereal products are currently widely recognized and understood because of the presence of a broad range of bioactive compounds [1]. Whole grain cereals are a rich source of carbohydrates, oils, proteins, vitamins, and minerals. The increasing demand for cereals and products thereof is reflected also by the fact that the world cereal production has reached its maximum level, exceeding 2.609 × 10^9^ tonnes in 2016, having increased steadily in the last 8 years [2]. Although it is estimated that it is possible to maintain present food consumption levels by increasing overall food supplies in quantitative terms, providing quality food that is nutritious and free from contaminants is becoming a very challenging task. Among food contaminants, mycotoxins can have serious consequences in terms of both human and animal health as well as huge economic impacts [3].

Mycotoxins are toxic products of secondary metabolism of microscopic filamentous fungi. These ubiquitous microorganisms are able to colonize various agricultural commodities either before harvest or under postharvest conditions, thus causing, in addition to mycotoxin contamination, a serious loss of harvest yield and quality of the infested commodity. According to the European Commission [4], it has been estimated that 5–10% of global production is lost annually because of mycotoxin contamination. However, in a recent survey on mycotoxin contamination, more than 80% of samples were contaminated with at least one mycotoxin and 45% contained more than one secondary metabolite of fungi [5]. Currently, there are approximately 100,000 described fungal species. The number of the most commonly occurring species in foods/feeds and indoor environments is estimated to be around 175 [6]. The most toxigenic species belong mainly to three fungi genera: *Fusarium*, *Aspergillus*, and *Penicillium* [7–10]. These fungi can produce a wide range of mycotoxins differing not only in their chemical structures but also in the mode of toxicological actions. However, the health risk to humans and animals is currently attributed to only a limited number of them. With regard to high incidences of contamination (and thus possible dietary exposures) and their toxicity, aflatoxins (AFs) (from *Aspergillus*), ochratoxins (from *Aspergillus* and *Penicillium*), trichothecenes, fumonisins B (FBs), and zearalenone (ZEN) (from *Fusarium*) are of greatest concern. Their toxic effects range from various effects on the liver, kidney, hematopoietic system, immune system, and fetal and reproductive systems to significantly contributing to carcinogenetic and mutagenic developments [11, 12]. The effect of mycotoxin exposure differs greatly. The susceptibility of animals and humans to the toxicological effects of mycotoxins differs with species, age, nutrition, length of exposure, and other factors. Evaluation of adverse health effects is complicated by exposure to various co-occurring mycotoxins, which may lead to additive, synergic, or antagonist toxic effects [13].

**Fig. 1:**
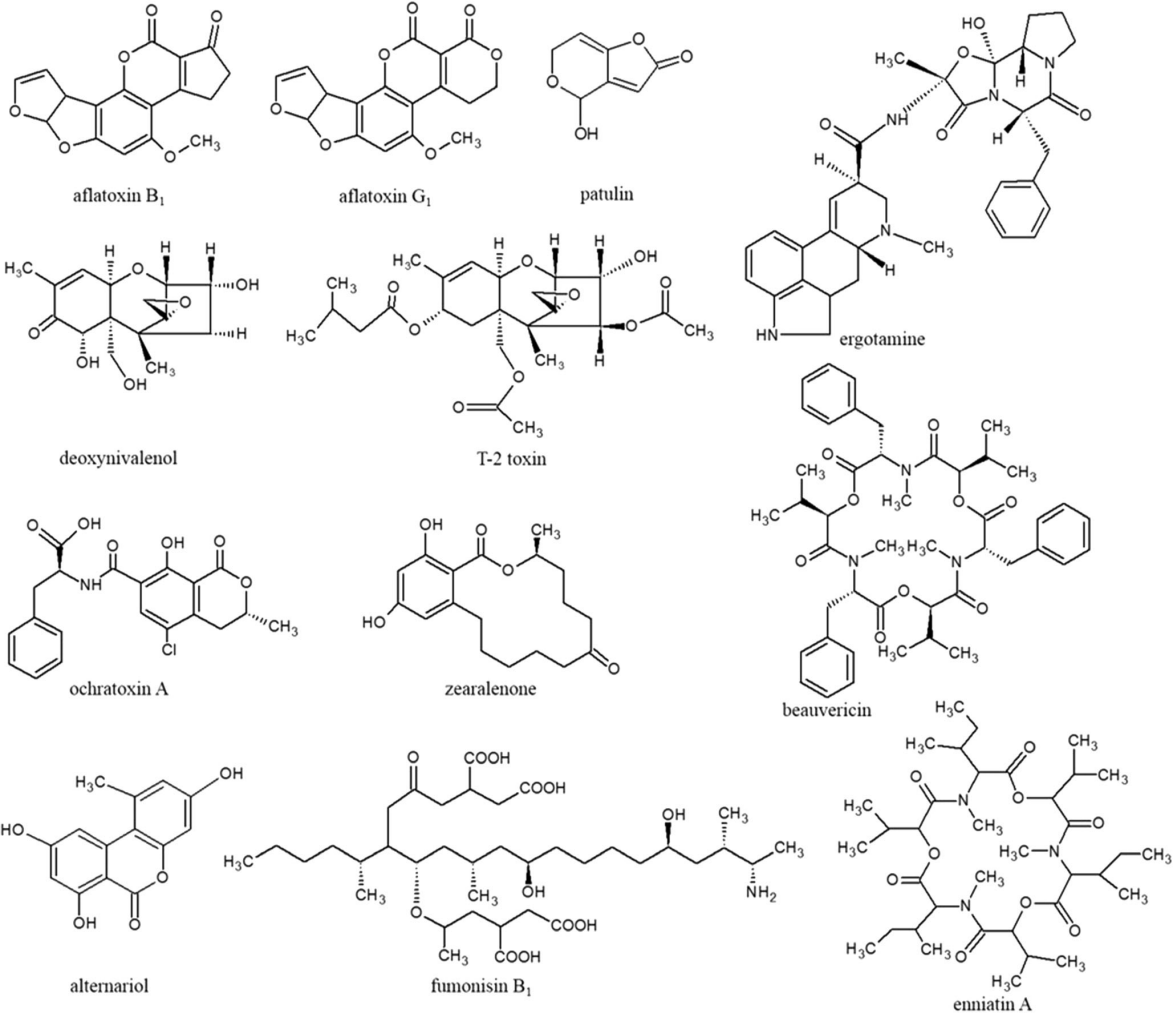
Chemical structures of the most important mycotoxins belonging to the group of *Aspergillus* toxins (aflatoxin B_1_, alfatoxin G_1_, ochratoxin A, patulin), *Fusarium* toxins (deoxynivalenol, T-2 toxin, zearalenone, fumonisin B_1_, beauvericin, enniatin A), *Alternaria* toxins (alternariol), ergot alkaloids (ergotamine), and *Penicillium* toxins (patulin, ochratoxin A)

With regard to the health hazards attributed to mycotoxins and their impact on consumers (and farm animals), many countries have set up regulations for their control in the food and feed chain. On the world scale, the Joint FAO/WHO Expert Committee on Food Additives has assessed the toxicity of various mycotoxins and related health risks. In the European Union (EU), scientific opinions on the risk assessment of mycotoxins have been issued by the European Food Safety Authority (EFSA), which advises the European Commission Directorate-General for Health and Food Safety. Currently, only maximum levels for AFs, deoxynivalenol (DON), ZEN, ochratoxin A (OTA), FBs, and patulin in various foodstuffs have been set in EU countries [14, 15]. The chemical structures of some EU-regulated mycotoxins and other toxicologically important mycotoxins are depicted in Fig. 1. The establishment and acceptance of new regulatory limits is a long-term and complex process consisting of the evaluation of occurrence data that are important for overall risk assessment, availability, and understanding of toxicological data. To obtain such information, advanced analytical methods are required in both research and official control laboratories for reliable and accurate determination of mycotoxins. The determination of mycotoxins in cereals and cereal-based products is a challenging task because of their low occurrence levels and the complexity of the matrices [13].

In the past, the analytical methods were more focused on routine analysis (i.e., mainly methods for a single analyte/matrix or groups of structurally related mycotoxins in a respective matrix had been developed). These methods were usually based on specific sample preparation protocols followed by traditional chromatographic separation. Primarily liquid chromatography (LC) coupled with ultraviolet/diode array detection and fluorescence detection, was used, but detection by mass spectrometry (MS) was rarely used. Gas chromatography with either electron capture detection or MS was used in routine determination of mycotoxins (e.g., trichothecenes) after time-consuming and laborious derivatization [16]. However, ongoing developments in the field of LC–MS technology have led to the availability of high-throughput instrumentation meeting the current demands of scientists and regulatory authorities for mycotoxin detection. The use of LC–MS in the determination of low molecular weight contaminants and residues at trace levels has significantly increased during the past two decades. Because of the unique features of this technique, LC–MS became a tool of choice to deal with a number of analytical challenges related to chemical food and feed safety testing in both research and routine commercial laboratories. In the field of mycotoxin determination there is a clear trend toward the use of multiple-analyte methods based on ultrahigh-performance LC (UHPLC) coupled with MS with various mass analyzers. LC–MS-based workflows provide significantly higher selectivity and sensitivity, increased confidence in the identification of analytes, and wider analyte/matrix scope as compared with traditional methods using conventional detectors [17]. LC–MS also facilitates the use of streamlined sample preparation procedures that save time and labor and reduce the overall costs associated with mycotoxin testing. State-of-the-art LC–MS methods for the determination of mycotoxins may largely differ in terms of analyte scope depending on the intended use of the resulting data. Although some of the available methods focus exclusively on mycotoxins with regulatory limits in place, others may allow (semi)quantitative or qualitative analysis of hundreds of mycotoxins, metabolites, or degradation products in a single analytical run. Having a large analyte scope usually results in the need for compromises in terms of the method performance characteristics achieved for the respective compounds. Besides targeted applications, LC–MS also plays an invaluable role in metabolomics-based studies dealing with the discovery and elucidation of new toxins or metabolites, as discussed in detail later in this review.

Currently, there are a wide range of LC-compatible MS instruments available on the market. A detailed overview and discussion of the advantages and disadvantages of current MS systems allowing measurements in low, medium and (ultra)high mass resolving power modes was provided in several recent comprehensive reviews [18, 19]. From the available studies, the MS detectors used in mycotoxin analysis include triple quadrupole (QqQ), ion trap, time-of-flight (TOF), and orbital ion trap mass analyzers, as well as hybrid systems that combine two types of analyzers. The latter group includes quadrupole–linear ion trap (QLIT), double quadrupole–TOF (QqTOF), quadrupole–orbital ion trap quadrupole–Orbitrap; Q–Orbitrap, and linear ion trap–orbital ion trap systems. The distribution of the LC–MS techniques applied in mycotoxin determination from 2012 to 2016 is depicted in Fig. 1.

In the past 10 years, several review articles on the occurrence and determination of mycotoxins have been published [11, 13, 16, 20–24]. Moreover, new developments and updates in this field are covered on a yearly basis in *World Mycotoxin Journal* [25–28].

This review thus provides insight into LC–MS-based methods for the determination of co-occurring mycotoxins. The aim is not to give a comprehensive overview of all published methods, but rather to focus on LC–tandem MS (MS/MS) targeted approaches and on untargeted analysis using LC–high-resolution MS (HRMS), including application of these approaches in the analysis of cereals and cereal-based food products. The advantages and limitations of both methods are critically assessed.

## Requirements and guidance for quantification and proper validation

Basic validation of an analytical method is a crucial part of the overall process of implementation of a new method. It must demonstrate that the analytical method complies with the criteria applicable for the relevant performance characteristics (i.e., it confirms that the method is suitable for the intended applications and provides reliable results). In the EU, only general guidelines on the performance of analytical methods and the interpretation of results are laid down in Commission Decision 2002/657/EC [29]. This document gives the specification of individual performance characteristics that have to be evaluated during method validation. According to the in-house validation approach, specificity, trueness, recovery, repeatability, reproducibility, decision limit (CCα), detection capability (CCβ), calibration curve (linearity), and ruggedness should be evaluated (Table 1). Strict guidelines on how to perform the experiments for the evaluation of the individual performance characteristics are also given here. Additionally, the term “confirmatory method” has been established. Any confirmatory method has to provide full information on the chemical structure of an analyte. Furthermore, an internal standard, preferably isotope labeled, should be used. Therefore, LC–MS/MS and LC–HRMS are recommended as the techniques of first choice.

**Table 1:** Overview of performance characteristics of an analytical method defined in Commission Decision 2002/657/EC

Performance characteristic	Definition
Accuracy	The closeness of agreement between a test result and the accepted reference value. It is determined by determining trueness and precision
Detection capability (CCβ)	The smallest content of the substance that may be detected, identified, and/or quantified in a sample with an error probability of *β*. For mycotoxins with no legislation limit, the detection capability is the lowest concentration at which a method is able to detect truly contaminated samples with a statistical certainty of 1-*β*. For mycotoxins with a legislation limit, the detection capability is the concentration at which the method is able to detect the legislation limit concentration with a statistical certainty of 1-*β*. *β* error means the probability that the tested sample is truly noncompliant, even though a compliant measurement has been obtained (false compliant decision)
Decision limit (CCα)	The limit at and above which it can be concluded with an error probability of *α* that a sample is noncompliant. *α* error means the probability that the tested sample is compliant, even though a noncompliant measurement has been obtained (false noncompliant decision)
Precision	The closeness of agreement between independent test results obtained under stipulated (predetermined) conditions. The measure of precision is usually expressed in terms of imprecision and computed as the standard deviation of the test results. Less precision is determined by larger standard deviation
Recovery	The percentage of the true concentration of a substance recovered during the analytical procedure. It is determined during validation if no certified reference material is available
Repeatability	The precision under repeatability conditions. Repeatability conditions means conditions where independent test results are obtained with the same method on identical test items in the same laboratory by the same operator using the same equipment
Reproducibility	The precision under reproducibility conditions. Reproducibility conditions means conditions where test results are obtained with the same method on identical test items in different laboratories with different operators using different equipment. Participation in ring trials is needed
Ruggedness	The susceptibility of an analytical method to changes in experimental conditions that can be expressed as a list of the sample materials, analytes, storage conditions, and environmental and/or sample preparation conditions under which the method can be applied as presented or with specified minor modifications. For all experimental conditions that could in practice be subject to fluctuation, any variations that could affect the analytical result should be indicated.
Specificity	The ability of a method to distinguish between the analyte being measured and other substances. This characteristic is predominantly a function of the measuring technique described, but can differ according to the class of the compound and the matrix
Trueness	The closeness of agreement between the average value obtained from a large series of test results and an accepted reference value. Trueness is usually expressed as bias

LC separation should be done with the appropriate LC column. The minimum acceptable retention time of the analyte of interest has to be at least twice the retention time corresponding to the dead volume of the column and has to match that of the calibration standard. The width of the retention time window should correspond to the resolving power of the chromatographic system. Moreover, the relative retention time of the analyte should match that of the calibration standard with a tolerance of ±2.5%.

MS detection should be done by use of MS techniques such as recording of full mass spectra or selected-ion monitoring (SIM), as well as MS/MS techniques such as selected-reaction monitoring (SRM). In HRMS the resolution should typically be greater than 10,000 for the entire mass range (according to the 10% valley definition [30]). In the full scan, the presence of all diagnostic ions (protonated and deprotonated molecules, characteristic fragment ions, and isotope ions) with a relative intensity of more than 10% in the reference spectrum of the calibration standard is obligatory. For SIM and SRM, a protonated or deprotonated molecule should preferably be one of the diagnostic ions selected. The diagnostic ions selected should not exclusively originate from the same part of the molecule. The signal-to-noise ratio for each diagnostic ion should be higher than 3:1. For the interpretation of data, a system of identification points should be used. In the case of mycotoxins, a minimum of three identification points are required per analyte. That means that two precursor ion to product ion transitions or one precursor ion and one product ion are required per analyte when low-resolution MS/MS or accurate mass HRMS is used, respectively [29].

The trueness of a quantitative confirmatory method has to be verified either by the repeated analysis of a certified reference material or, if this is not available, through the recovery of additions of a known amount of the analyte to a blank matrix. The analytical standards and certified reference materials for mycotoxins are supplied by two companies (Romer Labs and Sigma-Aldrich) [31, 32]. The guideline ranges for the deviation of the experimentally determined recovery corrected mean mass fraction from the certified value are -50% to +20% for mass fraction below 1 μg/kg, -30% to +10% for mass fraction between 1 and 10 μg/kg, and -20% to +10% for mass fraction above 10 μg/kg [29]. The precision of a quantitative confirmatory method expressed as the interlaboratory coefficient of variation (CV) for the repeated analysis of a reference or fortified material under reproducibility conditions (definition in Table 1) should not exceed the level calculated by the Horwitz equation: CV = 2^(1-0.5log*C*)^, where *C* is the mass fraction. For instance, the CV for a mass fraction of 100 μg/kg should not exceed 23% [29]. The trueness and precision of the analytical methods intended for the official control of mycotoxins are laid down in Commission Regulation (EC) No 401/2006 [33]. Participation in interlaboratory testing is an efficient tool to demonstrate a sufficient level of method trueness.

A drawback of both aforementioned European Commission decision and regulation is that the LC–MS methods counting more than 100 mycotoxins are not considered. Therefore, the performance of some validation experiments for such a high number of analytes is not feasible. For instance, to find a blank material free of a broad spectrum of mycotoxins is almost impossible, the definition of matrix effects and their evaluation is missing, the term “recovery” is not exactly specified, and the determination of the limit of detection (LOD) and limit of quantification (LOQ) by the spiking of 20 replicates at one level for various matrices is not feasible for hundreds of analytes because of the cost of analytical standards. Moreover, the availability of isotopically labeled standards (as it is recommended they be used) and certified reference materials on the market is also limited. Therefore, in practice, guidance document SANTE 11945/2015 [34] designed for multiresidue determination of pesticides was very useful also for validation of LC–MS methods for multiple determination of mycotoxins. Briefly, matrices are grouped on the basis of water/sugar/fat content, and for each group one representative matrix should be validated. Sensitivity, mean recovery (extraction efficiency), precision, and LOQ have to be evaluated in the method validation. The spiking experiments have to be performed on a minimum of five replicates at two different levels (a low level to check the sensitivity and a higher level). The method LOQ is defined as the lowest validated spiking level meeting the method performance acceptability criteria [mean recovery of 70–120% with a relative standard deviation (RSD) of 20% or less]. The criteria for LC–MS in this document do not differ from those specified in Commission Decision 2002/657/EC. To ensure the accuracy of the results generated, a quality control is recommended to be introduced in the laboratory. In practice, most of laboratories regularly participate in proficiency ring trials.

**Fig. 2:**
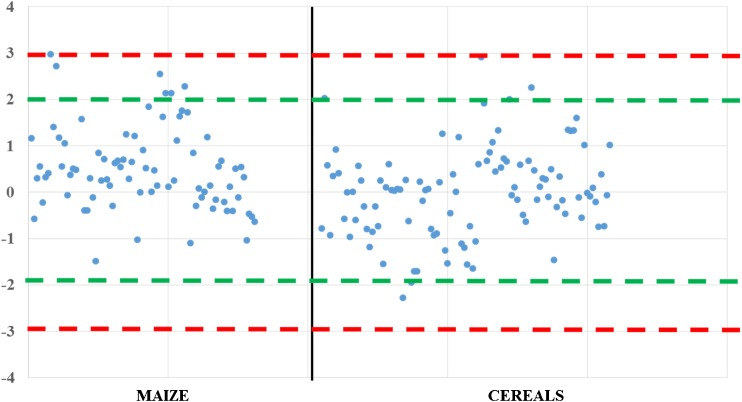
An example of the *z* score compilation obtained by the multimycotoxin liquid chromatography–tandem mass spectrometry method in proficiency testing organized by the Bureau Interprofessionnel des Études Analytique (BIPEA). Green lines borders of acceptable range of *z* scores, red lines borders of questionable range of *z* scores, area outside red lines unacceptable values

Proficiency testing is an effective procedure for quality assurance and performance verification in chemical analysis laboratories, ensuring that laboratory validation and within-laboratory procedures are working satisfactorily. The individual laboratory performance is expressed in terms of the *z* score in accordance with ISO 13525:2015 [35] and is calculated as *z* = (*χ*_lab_ - *χ*_assigned_)/*σ*_p_, where *χ*_lab_ is the mean of the two measurement results reported by a participant, *χ*_assigned_ is the assigned value (robust mean), and *σ*_p_ is the standard deviation for proficiency assessment derived from the truncated Horwitz equation. The *z* scores obtained are interpreted as follows: |*z*| ≤ 2 is an acceptable result, 2 < |*z*| ≤ 3 is considered a questionable result, and |*z*| > 3 is an unacceptable result. An example of the *z* scores obtained by the “dilute and shoot” multimycotoxin LC–MS/MS method [36] in routine proficiency testing organized by the Bureau Interprofessionnel des Études Analytique (BIPEA) is displayed in Fig. 2.

Currently, several proficiency testing schemes for mycotoxins are available in Europe, such as those from FAPAS (UK), BIPEA (France), Dienstleistung Lebensmittel Analytik (Germany), DUCARES (Netherlands), LGC Standards Proficiency Testing (UK), and Test Veritas (Italy). A detailed list of these schemes is available from [37]. However, most proficiency testing schemes organized by the aforementioned providers are focused on only a single mycotoxin or mycotoxins belonging to the same group. The first multimycotoxin proficiency testing scheme was organized by the Institute of Sciences of Food Production of the National Research Council of Italy in 2011. Since then, several other multimycotoxin proficiency testing schemes has been organized. The results of these trials have been summarized by De Girolamo et al. [38].

## LC–MS/MS-based approaches intended for the targeted determination of mycotoxins

**Fig. 3:**
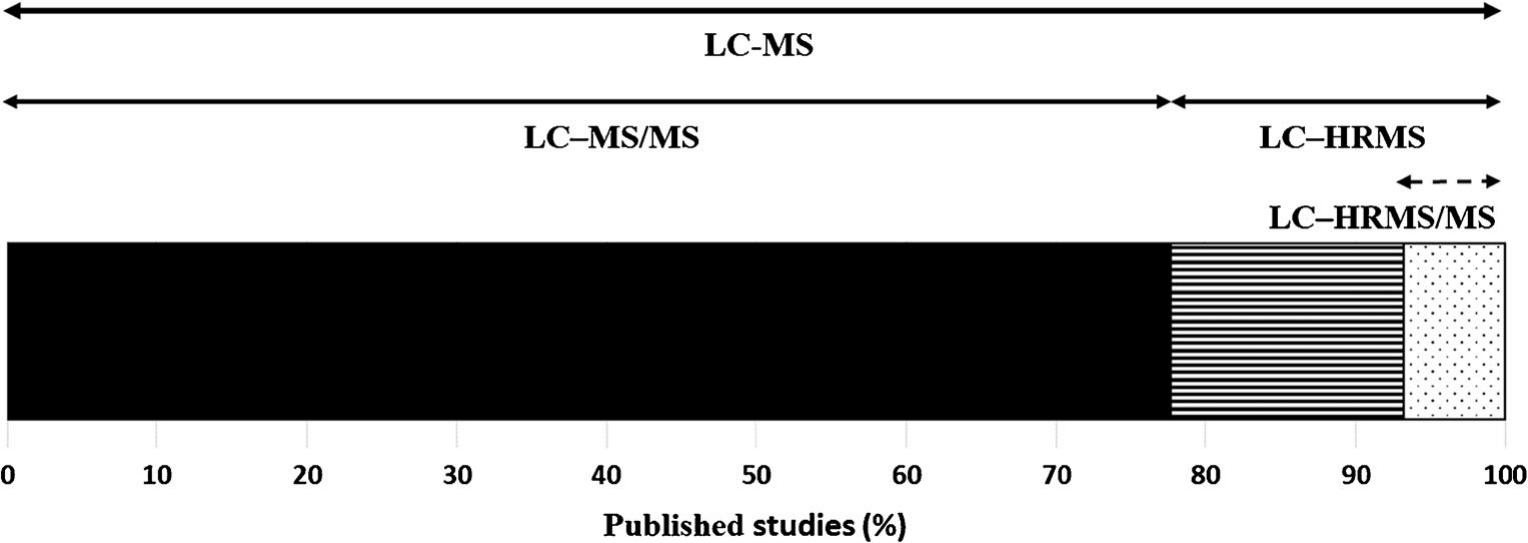
An overview of use of liquid chromatography (LC)–mass spectrometry (MS) instruments in studies focused on mycotoxin analysis published between 2012 and 2016. LC–MS/MS covers studies using instruments equipped with triple quadrupole, quadrupole–linear ion trap, and ion trap mass analyzers; LC–high resolution MS (HRMS)/MS covers studies using instruments equipped with quadrupole–time of flight or quadrupole–orbital ion trap mass analyzers

Almost 80% of all published LC–MS studies on mycotoxins since 2012 used methods based on LC–MS/MS (Fig. 3). The term “targeted” analysis implies that only “known” mycotoxins can be determined. In targeted mycotoxin determination, the complexity of the analyzed matrix and the range of “target mycotoxins” are the most important factors in determining a suitable instrument to be used for that particular application. A detailed description of the analytical methods discussed in the following text is given in Table 2.

**Table 2:** Detailed description of the setup of some liquid chromatography (LC)–mass spectrometry (MS) methods for mycotoxin determination

Reference	Extraction	Cleanup	Analytes	Matrix	LC–MS instrument	LC conditions	MS conditions
[39]	CH_3_CN–H_2_O (84:16, v/v)	MycoSep 226 AflaZON+, MycoSep 227 (both Romer Labs)	NIV, DON, FUS-X, 3ADON, 15ADON, DAS, HT2, T2, ZEN	Maize	QTRAP MS/MS instrument (Sciex) coupled to 1100 series LC system (Agilent Technologies)	Aquasil RP-18 column (100 mm × 4.6 mm, 3 μm) + C_18_ guard column; 25 °C, flow rate 1000 μL/min, injection volume 25 μL; eluent A H_2_O–CH_3_OH (80:20, v/v), eluent B H_2_O–CH_3_OH (10:90, v/v), both containing 5 mM NH_4_CH_3_COO^-^; gradient 0.5 min 0% eluent B, linear gradient to 100% eluent B to 4.5 min, 100% eluent B to 7 min, 7.1 min 0% eluent B, reequilibration 3 min, total run 10 min	APCI± MRM, monitoring of 2 transitions (1 quantifier and 1 qualifier), dwell time 100 ms, polarity switching (2 periods)
[40]	CH_3_CN–H_2_O (84:16, v/v)	MycoSep 226 AflaZON+	AFs, NIV, DON, 3ADON, 15ADON, FUS-X, HT2, T2, ZEN, OTA, STER, CIT, verruculogen	Various foods and feed	Quattro Ultima QqQ instrument (Micromass) coupled to Acquity UHPLC system (Waters)	UPLC BEH C_18_ (100 mm × 2.1 mm, 1.7 μm); 35 °C, flow rate 300 μL/min, injection volume 5 μL; eluent A ESI+ 10 mM NH_4_CH_3_COO^-^, ESI− 0.1% 0.1% (v/v) aqueous NH_3_, eluent B CH_3_OH; gradient initially 20% eluent B, linear increase to 5.5 to 85% eluent B, 100% eluent B within 0.3 min, reequilibration for 2 min at 20% eluent B, total run 10 min	ESI+, ESI−, MRM, 2 chromatographic runs, monitoring of 2 transitions (1 quantifier and 1 qualifier)
[41]	2-step extraction: (1) PBS; (2) 70% CH_3_OH	AOZFDT2 (VICAM)	AFs, OTA, FBs, DON, ZEA, T2, HT2	Maize	QTRAP MS/MS (Sciex) instrument coupled to 1100 micro LC system (Agilent Technologies)	Gemini C_18_ column (150 mm × 2 mm, 5 μm) + Gemini C_18_ guard column (4 mm × 2 mm, 5 μm); 40 °C, flow rate 200 μL/min, injection volume 20 μL; eluent A H_2_O, eluent B CH_3_OH, both containing 0.5% CH_3_COOH and 1 mM NH_4_CH_3_COO^-^; gradient 3 min at 20% eluent B, jump to 40% eluent B, linear increase to 63% eluent B within 35 min, 63% eluent B for 11 min, reequilibration at 20% eluent B for 10 min, total run 59 min	ESI+, ESI−, dMRM, monitoring of 2 transitions (1 quantifier and 1 qualifier), time window of 1 MRM 0.8 min, cycle time 0.55 s
[42]	CH_3_CN–H_2_O–CH_3_COOH (79.5:20:0.5, v/v/v). Evaporation and redissolution in PBS before IAC	Myco6in1+ (VICAM)	AFs, OTA, FBs, DON, ZEN, T2, HT2	Barley, maize breakfast cereals, peanuts	QTRAP 4500 instrument (Sciex) coupled to a Prominence UFLC XR chromatography system (Shimadzu)	Acquity UPLC HSS T3 end-capped C_18_ column (100 mm × 2.1 mm, 1.7 μm), 40 °C, flow rate 400 μL/min, injection volume 10 uL; eluents A H_2_O, eluent B CH_3_OH, both containing 5 mM NH_4_CH_3_COO^-^; gradient 5% eluent B increased to 50% eluent B in 1 min, linear increase to 100% eluent B within 6 min, 100% eluent B to 8 min, at 8.1 min initial conditions 5% eluent B, reequilibration at 5% eluent B for 2 min, total run 10 min	ESI± MRM, 2 periods, monitoring of 2 transitions (1 quantifier and 1 qualifier), dwell time 50 ms, polarity switching (2 periods)
[43]	2-step extraction: (1) H_2_O; (2) CH_3_OH. Evaporation and redissolution in PBS before IAC	Myco6in1+ (VICAM)	AFs, OTA, FBs, DON, ZEN, T2, HT2, NIV	Maize, durum wheat, corn flakes, maize crackers	QTRAP MS/MS instrument (Sciex) coupled to 1100 micro LC system (Agilent technologies)	Gemini C_18_ column (150 mm × 2 mm, 5 μm) + Gemini C18 guard column (4 mm × 2 mm, 5 μm); 40 °C, flow rate 200 μL/min, injection volume 20 μL; eluent A H_2_O, eluent B CH_3_OH, both containing 0.5% CH_3_COOH and 1 mM NH_4_CH_3_COO^-^; gradient 3 min at 20% eluent B, jump to 40% eluent B, linear increase to 63% eluent B within 35 min, 63% eluent B for 11 min, reequilibration at 20% eluent B for 10 min, total run 59 min	ESI± MRM, monitoring of 2 transitions (1 quantifier and 1 qualifier), dwell time 100 ms, polarity switching (2 periods)
[44]	NaCl + H_2_O–CH_3_OH (30:70, v/v). Dilution with PBS before IAC	OCHRAPREP + DZT MS-PREP, AOF MS-PREP + DZT MS-PREP, AFLAOCHRA PREP + DZT MS-PREP (R-Biopharm)	OTA + DON, ZEN, T2, HT2; AFs, FBs, OTA + DON, ZEN, T2, HT2; AFs, OTA + DON, ZEN, T2, HT2	Wholemeal bread, maize and maize-based products including infant foods, oat-based muesli	Acquity TQD tandem QqQ MS instrument (Waters)	Gemini C_18_ column (150 mm × 2 mm, 5 μm), 40 °C, flow rate 300 μL/min, injection volume 20 μL; eluent A H_2_O–CH_3_OH (95:5, v/v), eluent B H_2_O–CH_3_OH (98:3, v/v), both containing 0.5% HCOOH and 1 mM NH_4_HCOO^-^; gradient 20% eluent B for 0.1 min, to 10 min linear increase to 90% eluent B, 90% eluent B to 15 min, reequilibration at 20% eluent B, total run 20 min	ESI+ MRM, monitoring of 2 transitions (1 quantifier and 1 qualifier), 6 acquisition periods, dwell times from 0.1 to 0.27 s
[45]	QuEChERS		AFs, FBs, NIV, DON, 3ADON, 15ADON, FUS-X, HT2, T2, ZEN, OTA, DAS, NEO	Rice, corn, wheat, rye, oat, barley, infant cereals, soya, corn gluten	QTrap 4000 instrument (Sciex) coupled to 1100 series LC system (Agilent Technologies)	Zorbax Bonus-RP column (150 mm × 2.1 mm, 3.5 μm) + Zorbax RB C_8_ guard column (12.5 mm x 2.1 mm, 3.5 μm), flow rate 250 μL/min, injection volume 40 μL; eluent A 0.15% (v/v) HCOOH + 10 mM NH_4_HCOO^-^, eluent B 0.05% HCOOH (v/v) in CH_3_OH; gradient: 0% eluent B at 1 min, linear increase to 100% eluent B until 15 min, 100% eluent B for 5 min, reequilibration at 0% eluent B for 5 min, total run 25 min	ESI± MRM, monitoring of 2 transitions (1 quantifier and 1 qualifier), 3 acquisition periods
[46]	QuEChERS		AFs, FBs, DON, HT2, T2, ZEN, OTA	Wheat, maize, rice	Micromass Quattro Micro QqQ coupled to Alliance 2695 system (Waters)	Atlantis RP C_18_ column (150 mm × 2.1 mm, 5 μm), 30 0°C, flow rate 300 μL/min, injection volume 20 μL; eluent A H_2_O–CH_3_OH (90:10, v/v), eluent B H_2_O–CH_3_OH (10:90, v/v), both containing 5 mM NH_4_CH_3_COO^-^; gradient 20% eluent B for 0.1 min, until 10 min linear increase to 90% eluent B, 90% eluent B to 15 min, reequilibration at 20% eluent B, total run 20 min	ESI± MRM, monitoring of 2 transitions (1 quantifier and 1 qualifier), 3 acquisition periods
[47]	QuEChERS, dilute and shoot		38 mycotoxins and 288 pesticides	Apple baby food, wheat flour, paprika, black pepper, sunflower seed	QTRAP 5500 instrument (Sciex) coupled to Acquity UHPLC system (Waters)	Acquity UPLC HSS T3 end-capped C_18_ column (100 mm × 2.1 mm, 1.8 μm), 40 °C, flow rate 350–700 μL/min, injection volume 3 μL; ESI+ eluent A: H_2_O, eluent B: CH_3_OH, both containing 0.2% HCOOH + 5 mM NH_4_HCOO^-^; ESI− eluent A H_2_O, eluent B CH_3_OH, both containing 0.2% HCOOH + 5 mM NH_4_HCOO^-^; gradient: 10% eluent B with flow rate 350 μL/min increased to 50% eluent B in 1 min, linear increase to 100% eluent B within 10 min and simultaneous increase of flow rate to 550 μL/min, flow rate 0.7 μL/min at 100% eluent B, reequilibration for 2.5 min at 10% eluent B at 450 μL/min, total run 15.5 min	ESI+, ESI−, dMRM, time window for 1 MRM 0.8 min, cycle time 0.55 s
[48]	Dilute and shoot	No	39 mycotoxins	Wheat, maize	QTRAP 4000 instrument (Sciex) coupled to 1100 series LC system (Agilent Technologies)	Gemini C_18_ column (150 mm × 2 mm, 5 μm) + Gemini C_18_ guard column (4 mm × 2 mm, 5 μm); 40 °C, flow rate 1000 μL/min, injection volume 5 μL; eluent A CH_3_OH–H_2_O–CH_3_COOH (10:89:1, v/v/v), eluent B CH_3_OH–H_2_O–CH_3_COOH (97:2:1, v/v/v), both containing 5 mM NH_4_CH_3_COO^-^; gradient 2 min at 100% eluent A, linear increase to 100% eluent B within 12 min, held at 100% eluent B for 3 min, reequilibration at 100% eluent A for 4 min, total run 19 min	ESI+, ESI−, dMRM, dwell time 100 ms, pause time 5 ms
[36]	Dilute and shoot	No	295 analytes	Apple puree, hazelnut, maize, green pepper	QTRAP 5500 instrument (Sciex) coupled to 1290 series LC system (Agilent Technologies)	Gemini C_18_ column (150 mm × 2 mm, 5 μm) + Gemini C_18_ guard column (4 mm × 2 mm, 5 μm); 40 °C, flow rate 1000 μL/min, injection volume 5 μL; eluent A CH_3_OH–H_2_O–CH_3_COOH (10:89:1, v/v/v), eluent B CH_3_OH–H_2_O–CH_3_COOH (97:2:1, v/v/v), both containing 5 mM NH_4_CH_3_COO^-^; gradient: 2 min at 100% eluent A, linear increase to 50% eluent B within 3 min, linear increase zo 100% eluent B within 9 min, hold at 100% eluent B for 4 min, reequilibration at 100% eluent A for 2.5 min, total run 20.5 min	ESI+, ESI−, dMRM, MRM window ±27 s for positive mode, ±42 s for negative mode, scan time 1 s
[49]	Raw extract, SIDA	No	AFs, FBs, DON, HT2, T2, OTA, ZEN	Maize, cereal-based products	6490 triple-quadrupole instrument coupled to 1290 series UHPLC system (both Agilent Technologies)	Zorbax RRHL Eclipse Plus C_18_ column (100 mm × 2.1 mm, 1.8 μm); 30 °C, flow rate 350 μL/min, injection volume 3 μL; eluent A H_2_O–HCOOH (99.9:0.1, v/v), eluent B CH_3_OH–HCOOH (99.9:0.1, v/v) both containing 5 mM NH_4_HCOO^-^; gradient: 0.5 min at 30% eluent B, linear increase to 100% eluent B in 7.5 min, hold at 100% eluent B for 1.5 min, at 9.6 min back to 30% eluent B, reequilibration at 30% eluent B for 2 min, total run 11.5 min	ESI±, dMRM, monitoring of 2 transitions (1 quantifier and 1 qualifier)
[50]	CH_3_CN–H_2_O (84:16, v/v), SIDA	Bond Elut Mycotoxin SPE cartridges (Agilent Technologies)	NIV, DON, FUS-X, DON-3-Glc, 3ADON, 15ADON, HT2, T2, ENNs, BEA, ZEN	Barley, malt, oat, wheat, maize	QTRAP 4000 instrument (Sciex) coupled to LC-20A Prominence system series LC system (Shimadzu)	Hydrosphere RP-C18 column (100 mm × 3 mm, 3 μm) + C_18_ guard column; 40 °C, flow rate 200 μL/min, injection volume 10 μL; eluent A H_2_O–HCOOH (99.9:0.1, v/v), eluent B CH_3_OH–HCOOH (99.9:0.1, v/v); gradient ESI− 2 min at 10% eluent B, linear increase to 99% eluent B in 6 min, hold at 99% eluent B for 7.5 min, for 2 min back to 10% eluent B, reequilibration at 10% eluent B for 9.5 min, total run 25 min; ESI+ 2 min at 10% eluent B, linear increase to 87% eluent B in 6 min, hold at 87% eluent B for 7 min, increase to 100% eluent B in 5 min, hold at 100% eluent B for 3.5 min, for 2 min back to 10% eluent B, reequilibration at 10% eluent B for 9.5 min, total run 34.5 min	ESI−, ESI+, dMRM, 2 single chromatographic runs, monitoring of 2 transitions (1 quantifier and 1 qualifier)
[51]	CH_3_CN–H_2_O (84:16, v/v), evaporation, reconstitution in CH_3_OH and H_2_O	SPE (Oasis HLB columns)	AFs, OTA, DON, ZEN, T2, HT2	Wheat flour, barley flour, crisp bread	Accela HPLC system, Exactive HRMS instrument (Thermo Fisher Scientific); 1100 micro-LC system (Agilent Technologies), QTRAP instrument (Applied Biosystems)	Kinetex C_18_ column (100 mm × 2.1 mm, 2.6 μm); 40 °C, flow rate 200 μL/min, injection volume 20 μL; eluent A H_2_O, eluent B CH_3_OH, both containing 0.5% CH_3_COOH and 1 mM NH_4_CH_3_COO^-^; gradient 10% eluent B start, until 4 min linear increase to 40% eluent B, 60% eluent B in 27 min, keep for 5 min, reequilibration at 10% eluent B for 7 min, total run 20 min	HESI-II (heated-electrospray, ESI+, HCD fragmentation (in-source fragmentation)
[52]	QuEChERS (2 g sample, 10 mL 0.1% HCOOH in H_2_O, 3 min shaking, 10 mL CH_3_CN, 3 min shaking, 4 g MgSO_4_, 1 g NaCl, shaking)	No additional cleanup	3ADON, 15ADON, DON, DON-3-Glc, FUS-X, NIV, HT2, T2, DAS, NEO, AFs, OTA, FBs, STER, ZEN, penitrem A, BEA, *Alternaria* toxins, ergot alkaloids	barley	Accela HPLC system, Exactive HRMS instrument (Thermo Fisher Scientific)	Acquity UPLC HSS T3 column (100 mm × 2.1 mm, 1.8 μm); 40 °C, flow rate 300 μL/min, injection volume 5 μL; eluent A H_2_O with 5 mM NH_4_HCOO^-^ and 0.1% HCOOH, eluent B CH_3_OH; gradient: start with 5% eluent B, increase to 50% eluent B in 6 min, increase to 95% eluent B within 4 min, keep until 15 min of the run, reequilibration at 5% eluent B for 3 min	HESI-II, ESI+/ESI−
[53]	QuEChERS (2 g sample, 10 mL 0.1% HCOOH in H_2_O, 3 min shaking, 10 mL CH_3_CN, 3 min shaking, 4 g MgSO_4_, 1 g NaCl, 0.5 trisodium citrate dihydrate, shaking)	No additional cleanup	3ADON,15ADON, DON, DON-3-Glc, FUS-X, NIV, HT2, T2, DAS, NEO, AFs, OTA, FBs, STER, ZEN, mycophenolic acid, MON, BEA, *Alternaria* toxins, ergot alkaloids, culmorins	malting barley	Acquity UHPLC system (Waters), Q-Exactive system (Thermo Fisher Scientific)	Atlantis T3 column (100 mm × 2.1 mm, 3 μm); 30^o^C, flow rate 300 μL/min, injection volume 2 μL; eluent A CH_3_CN–H_2_O–CH_3_COOH (95:4.9:0.1, v/v/v), eluent B H_2_O–CH_3_COOH (99.9:0.1, v/v), both containing 5 mM NH_4_CH_3_COO^-^; gradient 5% eluent A start for 1 min, increase to 15% eluent A in 14 min, increase to 100% eluent A in next 15 min, kept at 100% eluent A for 3 min, reequilibration for 4.4 min	HESI-II (positive, negative)

*15ADON* 15-acetyldeoxynivalenol, *3ADON* 3-acetyldeoxynivalenol, *AFs* aflatoxins, *APCI* atmospheric pressure chemical ionization, *BEA* beauvericin, *CIT* citrinin, *DON-3-Glc* deoxynivalenol 3-glucoside, *DAS* diacetoxyscirpenol, *dMRM* dynamic multiple-reaction monitoring, *DON* deoxynivalenol, *ENNs* enniatins, *ESI* electrospray ionization, *FBs* fumonisins B, *FUS-X* fusarenon X, *HCD* high-energy collisional dissociation, *HPLC* high-performance liquid chromatography, *HRMS* high-resolution mass specrometry, *HT2* HT-2 toxin, *IAC* immunoaffinity chromatography, *MON* moniliformin, *MRM* multiple-reaction monitoring, *NEO* neosolaniol, *NIV* nivalenol, *OTA* typo in ocratoxin A, *PBS* phosphate-buffered saline, *QqQ* triple quadrupol, *QuEChERS* quick, easy, cheap, effective, rugged, and safe, *SIDA* stable isotope dilution assay, *SPE* solid-phase extraction, *STER* sterigmatocystin, *T2* T-2 toxin, *UFLC* ultrafast liquid chromatography, *UHPLC* ultrahigh-performance liquid chromatography, *UPLC* ultraperformance liquid chromatography, *ZEA* zearalanol, *ZEN* zearalenone

In MS/MS, the generation of analyte ions is followed by the selection of suitable precursor ions, and collision-induced dissociation (fragmentation) to form product ions that are further detected. LC–MS/MS techniques using a QqQ (and QLIT) mass spectrometer are the most frequently used approach in targeted mycotoxin determination. QqQ systems are typically operated in multiple-reaction monitoring (MRM) mode based on recording two or more precursor ion to product ion transitions. In contrast to the poor performance in full-scan mode, the use of MRM data acquisition provides a significant gain in both sensitivity and selectivity. The excellent sensitivity of QqQ analyzers when operating in SRM mode makes it possible to achieve low microgram per kilogram detection levels [54]. The average number of mycotoxins cited in the most recently published studies was about 30. Such methods cover a range of well-established mycotoxins, for which analytical standards are available (e.g., trichothecenes, enniatins, AFs B, *Alternaria* toxins, FBs, and ergot alkaloids). The matrices of interest are typically cereals, nuts, seeds, or baby food, but also more complex and problematic matrices such as spices, fruits, herbs, or food supplements are tested. Although MS/MS is generally considered to be a very selective technique especially for problematic matrices, in the case of challenging matrices the MS/MS signal might be overestimated and lost because of the complexity of some samples, which can finally result in false positive findings. In LC–MS methods, positive-mode electrospray ionization (ESI) is almost exclusively used to couple high-performance LC (HPLC) or UHPLC and MS detection. In theory, QqQ mass spectrometers can simultaneously detect a large number of targeted analytes. However, to achieve low LOQs compliant with the regulatory level and have an acceptable number of points per chromatographic peak to ensure accurate and reproducible quantification, the number of simultaneously recorded MRM transitions is limited. This is because of the need for sufficient dwell times for the recording of the respective MRM transitions. Faster instrument electronics and improved design of the collision cell significantly shorten the minimum dwell times that need to be used for each precursor ion–product ion pair monitored. The rapid multimethods that fully exploit the potential of the state-of-the-art QqQ/QLIT instruments are capable of the simultaneous analysis of up to 300 mycotoxins, their metabolites, or other related food contaminants depending on the length of the chromatographic run [36, 49, 55]. Such multimycotoxin methods are considered to be semiquantitative methods, since no pure analytical standards are available on the market and only in-house purified standards are used for quantification. This brings up a significant disadvantage associated with the use of QqQ MS detectors that do not allow nontargeted analysis (e.g., mycotoxin metabolites), as the detection conditions need to be typically optimized for each analyte with the use of a standard.

The main difficulty in LC–MS analysis of complex matrices is matrix effects. Components of the sample matrix can cause suppression (in most cases) or enhancement of the analyte signal during the ionization process and thus affect accurate quantification of analytes, leading to incorrect results, when pure solvent standards are used. A review dealing with matrix effects in LC–MS/MS methods was published in 2010 [56]. Matrix effects are a complex phenomenon. Their extent is dependent on many factors. First, the chemical structure and polarity of the analyte of interest play an important role. Furthermore, the matrix type and the relative concentrations of the components competing for the charges in the MS interface are also significant. Therefore, the entire sample preparation procedure and the chromatographic and MS conditions have to be optimized to decrease the matrix effects to a minimum [56]. Ion suppression might be caused by the presence of matrix compounds that are co-eluted with the target analytes and that reduce the ionization efficiency as well as affect the reproducibility and accuracy of the method [57]. Huge differences in the extent of matrix effects are not only seen for different matrices; high deviations between individual samples of one matrix type are also observed [58, 59]. Matrix effects were present also after highly extensive immunoaffinity cleanup [41, 42].

Standard addition is often used in routine analysis. It is applied in single-analyte methods rather than in multiple-analyte determination as it is laborious and costly because of the high consumption of analytical standards and double the number of LC runs [60]. The last and currently most frequently used approach is isotopically labeled internal calibration. Stable isotopically labeled standards share the same chemical and physical properties as the target analytes, but are still distinct by their different molecular masses. Additionally, they are not present in naturally contaminated samples. The principle of the use of isotopically labeled standards is that of the dilution of naturally abundant isotopic distribution. Therefore, this procedure is called “stable isotope dilution assay” (SIDA). The basic SIDA principle is to transfer the concentration of the analyte into an isotopologue ratio, which has to be stable during all analytical steps. Therefore, the first prerequisite for an internal standard is stable labeling. As carbon–carbon and nitrogen–carbon bonds are very unlikely to be cleaved, mainly labels consisting ^13^C and ^15^N are used for preparation of the isotopically labeled standards for mycotoxins. In contrast, losses of ^18^O and ^2^H can occur if these labels are at labile positions. For instance, ^18^O in carboxyl moieties can be exchanged in acidic and basic solutions. As a concern, deuterium (^2^H) is susceptible to “protium–deuterium exchange” if it is activated by an adjacent carbonyl group or aromatic systems. Moreover, chromatographic shifts caused by the isotope effect are more common in the case of deuterated standards than in the case of ^13^C- and ^15^N-labeled ones. Therefore, ^13^O and ^2^H labeling are less common in mycotoxin quantification using SIDA [61].

The following paragraphs highlight examples of currently used approaches for LC–MS/MS determination of mycotoxins in cereals and products thereof. The methods range from those intended for official control to multiclass methods used in research for both screening and quantitative purposes. In addition, how to cope with determination of conjugated mycotoxins will also be part of this section.

Most of the methods discussed have been developed for the determination of EU-regulated mycotoxins in various matrices to fulfill the strict requirements of EU legislation (e.g., low LODs for AFB_1_ and OTA in processed cereal-based food and baby food for infants and young children) [42, 44, 62, 63]. Therefore, to minimize the amount of undesirable matrix co-extracts (further discussed later), purification steps are usually required during sample preparation, especially in the case of complex matrices such as cereal-based products. Typical cleanup strategies involve commercially available solid-phase extraction (SPE) cartridges or immunoaffinity columns (IACs). However, despite the use of highly sensitive LC–MS instrumentation, achieving trace detection levels of some analytes in more than 200 multidetection methods is impossible when compromises have to be made with regard to both sample preparation and LC–MS/MS conditions. These methods rely on the injection of raw extracts [43, 64], “dilute and shoot” approaches [36, 47], or different modifications of the “quick, easy, cheap, effective, rugged, and safe” (QuEChERS) approach [48, 62, 64, 65].

### Strategies to eliminate matrix effects in (multi)mycotoxin determination

#### Solid-phase extraction

Cleanup techniques in mycotoxin analysis were last critically reviewed in 2008 [16]. For multitoxin analysis, mainly SPE cartridges are used. Nowadays, there are plenty of these from different manufacturers available on the market. For example, multifunctional columns, MycoSep^®^, containing a mixture of charcoal, ion-exchange resins, and other exchange materials are available for various combinations of mycotoxins or single mycotoxins [OTA, moniliformin, nivalenol (NIV)] [66]. Use of MycoSep^®^ columns has become a routine purification step especially for trichothecenes since the 1990s [66, 67]. The procedure involves extraction with aqueous acetonitrile and passing the extract through the SPE column, and preconcentration of the analytes of interest. Matrix co-extracts are retained on the column packing, and the analytes of interest pass to the supernatant. No washing step is required. One of the first pioneering LC–MS/MS methods using MycoSep^®^ 226 for purification of maize samples before simultaneous detection of *Fusarium* mycotoxins [NIV, DON, fusarenon X (FUS-X), 3-acetyldeoxynivalenol (3ADON), 15-acetyldeoxynivalenol (15ADON), diacetoxyscirpenol, HT-2 toxin (HT2), T-2 toxin (T2), and ZEN] was published in 2005 [39]. Although MycoSep^®^ 227 was also tested and satisfactory recoveries were obtained for most of the trichothecenes, 50% retention of NIV and complete retention of ZEN on the column packing was observed. MycoSep^®^ 226, designed for a broader range of polarities, increased the recovery rate for ZEN. However, more matrix components that also passed through the column caused high signal suppression, which resulted in a recovery of 30% for ZEN. Therefore, the addition of an internal standard (zearalanol) was used for ZEN quantification. The low recovery (50%) obtained for NIV was within the manufacturer’s specification. The method performance characteristics summarized in the electronic supplementary material show that because of MycoSep-based purification, low LODs can be achieved without highly sensitive state-of-the-art LC–MS/MS systems having to be used. An in-house validation of another method including MycoSep^®^ 226 was done for 17 mycotoxins, including AFs and trichothecenes, in various foods and feed [40]. Special attention was paid to the selection of proper eluents, the chromatographic column, and MS/MS conditions to achieve the highest possible sensitivity. The LOQs below 0.01 μg/kg for AFs, low intraday and interday precision (RSD below 10%), and high recoveries (70–110%) (see the electronic supplementary material) achieved passed the criteria of the official methods for mycotoxin determination in baby food [40].

Recently, MycoSpin^TM^ 400 for multimycotoxin LC–MS/MS analysis (optimized for most of the EU-regulated mycotoxins) in a spin format became available. So far, it has been applied to the purification of maize-based silages [68]. The manufacturer claims that highly accurate results are achieved by use of ^13^C-labeled standards [66]. Dispersive magnetic SPE might be an attractive technique as an alternative to classic SPE in the future [96]. However, compared with a classic on-column SPE, dispersive magnetic SPE requires the same time for a single extraction. The mechanism occurring in magnetic SPE is analogous to that in classic on-column SPE. The dispersion of the magnetic nanoparticles into the solution containing mycotoxins ensures a continuous and dynamic contact with the adsorbent surface, leading to more efficient analyte retention. The separation of the magnetic material with the adsorbed analytes from the solution is then realized by application of a magnet outside the vessel, avoiding centrifugation or filtration steps. Finally, after washing, analytes are eluted from the magnetic material by a proper solvent mixture. So far, only one study on the use of dispersive magnetic SPE in mycotoxin determination (AFs, ZEN, and OTA) in cereals has been published [96].

#### Immunoaffinity columns

IACs provide an even more specific cleanup compared with SPE cartridges. The principle is based on antibodies that entrap an analyte of interest. The matrix co-extracts are removed by washing, and “pure analyte” is released from the antibody with use of an organic solvent. IAC cleanup should provide a purified sample completely free of the matrix, which allows the use of a solvent calibration curve for quantification. However, as discussed later [41, 42], ion suppression/enhancement (caused by the IAC sorbent) has been observed. In the past, IACs were mainly designed for one or two toxins. However, legislative requirements led to the development of IACs for multiple regulated toxins; for example, AFLAOCHRA PREP^®^ (AFs and OTA), AO ZON PREP^®^ (AFs, OTA, and ZEN), and DZT MS-PREP^®^ (DON, ZEN, HT2, and T2). The only IAC that covers most of the EU-regulated mycotoxins is Myco6in1^+^ (VICAM) containing antibodies for AFs, OTA, FBs, DON, ZEN, NIV, T2, and HT2. Although, the application of IACs is a preferred and selective tool for sample cleanup, the sample extraction and handling procedure recommended by the manufacturer is very laborious and time-consuming, and produces large volumes of solvent waste. Special attention has to be paid to the extraction solvent, elution rate, and column capacity to achieve optimal recoveries of targeted analytes [41, 42]. In addition, column capacity may be hindered as a result of antibody cross-reactivity (affinity for other structurally related toxins). This, on the other hand, is a big advantage in the determination of conjugated mycotoxins (discussed later). An LC–MS/MS method for simultaneous determination of AFs, OTA, and *Fusarium* mycotoxins in maize using multifunctional IACs (AOZFDT2^TM^) was developed and validated in-house [41]. Despite the laborious cleanup and long chromatographic gradient (59 min), matrix effects were observed (i.e., significant suppression for AFB_1_ and AFG_1_ and a slight suppression for OTA). Although no matrix effects were observed for DON, T2, HT2, FBs, and ZEN, the study authors decided to use matrix-matched standards for quantification. When validated for maize, at EU maximum permitted levels, the method showed satisfactory performance in terms of recovery and repeatability (see the electronic supplementary material). The application of these IACs in analysis of maize-based cereals, barley, and peanuts was documented in another study [42]. The sample preparation procedure was markedly simplified, and the compatibility of several extraction solvents with the IACs was tested. Assessment of matrix effects confirmed the ion suppression observed for AFs and OTA [41] and showed significant ion enhancement for FBs. The follow-up evaluation on the “IAC solvent” calibration curve (when standards were prepared in solvent obtained from passing through the IAC) revealed that the ion suppression/enhancement was mainly caused by compounds flushed from the IAC packing. Therefore, “IAC solvent” standards can be used for quantification instead of matrix-matched calibration, and so this approach allows the use of one calibration for various matrices [42].

Other measures were taken to enhance the method; for example, changing from HPLC to UHPLC decreased the chromatographic run time from 59 to 10 min. Similarly, the sample preparation procedure [41] was simplified, and the modified method was validated for maize, durum wheat, corn flakes, and maize crackers [43]. Full in-house validation was performed for 12 mycotoxins at three levels (see the electronic supplementary material). Another option in determining multiple mycotoxins using IAC cleanup more efficiently is to use different IACs in tandem (i.e., the first column is connected below the glass vessel and the second column is connected below the first one). Thus sample loading, washing, and elution is achieved for two columns simultaneously. The use of columns in tandem offers a range of desired combinations of mycotoxins in accordance with EU regulations, so analytical cleanup can focus on target mycotoxins known to co-occur in specific matrices. OCHRAPREP^®^ and DZT MS-PREP^®^ columns have been used in tandem for analysis of wholemeal bread, AOF MS-PREP^®^ and DZT MS-PREP^®^ IACs have been combined for analysis of a range of maize and maize-based products, including infant foods, and AFLAOCHRA PREP^®^ and DZT MS-PREP^®^ columns have been combined for the analysis of oat-based muesli containing dried fruit and nuts [44].

In summary, immunoaffinity chromatography is still the most powerful cleanup method for up to six mycotoxins especially when used before LC-based (no MS) detection [62, 63]. Because of recent trends leading to the use of LC–MS/MS, it is no longer necessary in laboratories with sophisticated instrumentation. However, IACs are often used in LC–MS/MS when low LODs are required (e.g., OTA and AFB_1_ in baby food) [64].

#### LC–MS/MS with limited cleanup or without cleanup

The availability of sensitive LC–MS instruments that are less prone to matrix effects has led to the development and application of so-called multiple-analyte approaches. Hence, a clear trend toward the determination of a range of different mycotoxin classes can be observed, both in the literature and in research and routine laboratories. Because of the broad physicochemical properties of mycotoxins, a compromise, using nonspecific, minimal cleanup (if needed), has to be used to avoid a discrimination of some analytes during sample preparation processing.

##### QuEChERS approach

As a minimal cleanup, the QuEChERS approach is being used in multiclass analysis [69]. This approach was developed for multipesticide analysis, for very fast extraction and purification. The key principle is the partitioning of an acetonitrile–water mixture induced by addition of inorganic salts. While the analytes are largely transferred into an organic phase, more polar matrix impurities are left in an aqueous layer. Nevertheless, use of such a basic cleanup for multiresidue analysis in complex matrices leads inevitably to matrix effects, and thus affects sensitivity adversely. For this reason, LC–MS/MS methods including QuEChERS-based protocols are generally inefficient for the detection of AFs and OTA in baby foods at the EU limits. Hence, for these specific metabolites in baby foods, a multiresidue approach is often abandoned in favor of dedicated methods making use of specific cleanup with IACs [64] or a combination with another cleanup technique is used [41, 42, 45, 46, 64].

The original QuEChERS method for determination of pesticide residues consists of several steps: (1) extraction with acetonitrile, (2) partitioning step with magnesium sulfate (MgSO_4_) and sodium chloride (NaCl), (3) addition of an internal standard, (4) dispersive SPE—aliquot purified with MgSO_4_ and SPE sorbents [e.g., primary–secondary amine (PSA) salts, C_18_, C_8_], and (5) addition of an analyte protectant and adjustment of the pH. The implementation of the QuEChERS method in mycotoxin determination required some modifications depending on the spectrum of the analytes and the character of the matrix. For instance, PSA used for the removal of polar matrix components caused significant loss of FBs [45], and/or addition of water before acetonitrile extraction was needed for low water content matrices to increase the recovery yields [45, 47].

The QuEChERS-like approach was used for the development of an LC–MS/MS method for 17 mycotoxins in cereals for human consumption and infant cereals [45]. Besides the aforementioned modifications, the extraction was done with acidified acetonitrile to increase recoveries for FBs. Direct analysis of the extract after the partitioning step resulted in significant matrix effects, and thus insufficient sensitivity (especially for AFs). Several dispersive solid-phase extraction (SPE) sorbents were tested (SPE, Oasis HLB, Carbograph 4, C_18_, or dispersive SPE with both PSA and C_18_ modified silica gel). Finally, a simple defatting step with *n*-hexane followed by a two-step sequential reconstitution in aqueous methanol was shown to be the best adaptation for all analyte–matrix combinations. Although the performance characteristics of the method fulfilled the EU legislation criteria [33], it is not appropriate for official control of infant cereals. The maximum EU legislation level for AFB_1_ is 0.1 μg/kg but the LOQ was 1 μg/kg. The current method was applied in the analysis of more matrices (cereals, cocoa, oil, spices, infant formula, coffee, and nuts) and validated. Matrix effects were successfully corrected by ^13^C-labeled standards. To achieve lower LOQs for AFs and OTA in baby food, an additional cleanup step (immunoaffinity chromatography) was applied [64]. Positive identification of mycotoxins in the matrix was conducted according to the confirmation criteria defined in Commission Decision 2002/657/EC [29], while quantification was performed by isotopic dilution using ^13^C-labeled mycotoxins as internal standards. The LOQs were at or below the maximum levels set by Commission Regulation (EC) No 1881/2006 [14] for all regulated mycotoxin–matrix combinations. In particular, the inclusion of an immunoaffinity chromatography step allowed LOQs as low as 0.05 and 0.25 μg/kg to be achieved in cereals for AFs and OTA, respectively [64].

Another QuEChERS modification followed by LC–ESI-MS/MS was introduced for the determination of EU-regulated mycotoxins in wheat, maize, and rice [46]. Water soaking and acidified acetonitrile extraction with a mixture of magnesium sulfate, sodium chloride, sodium citrate tribasic dihydrate, and sodium citrate dibasic sesquihydrate (4:1:1:0.5) was followed by dispersive SPE with a mixture of magnesium sulfate and C_18_ sorbent. Purified extract was evaporated and reconstituted in aqueous methanol. The validation data obtained are summarized in the electronic supplementary material. It is worth noting that the greatest matrix effects were observed for maize.

##### “Dilute and shoot” approach

“Dilute and shoot” and the injection of raw extract, without any cleanup, is now commonly performed in multiresidue LC–MS analysis [47, 48, 65]. The dilute and shoot LC–MS/MS-based method is considered a pioneering method in the field of multimycotoxin determination [48]. Simple extraction with a mixture of acetonitrile–water–acetic acid (79:20:1, v/v/v) further diluted 1:1 with acetonitrile–water–acetic acid (20:79:1, v/v/v) was used in the determination of 39 mycotoxins, including conjugated metabolites, in cereals. Although the MS/MS parameters for most of the analytes could have been optimized in both polarities (i.e., analytes give the MS/MS signal in negative ionization mode as well as in positive ionization mode), some analytes (moniliformin, NIV, ZEN 14-glucoside) gave no or very weak signals in the positive mode. Because of the high number of analytes, the determination was performed in two chromatographic runs (positive and negative) to avoid losses in sensitivity caused by polarity switching. The method was validated for wheat and maize. Ion suppression effects caused by co-eluted matrix components were negligible in the case of wheat, whereas significant signal suppression for 12 analytes was observed in maize. The apparent recoveries were within the range of (100 ± 10)% for half of the analytes; in extreme cases the apparent recovery dropped to 20%. As an example of an analyte with low apparent recovery, FB_1_ (34%) can be given. Apparent recovery of 113% was observed for a common contaminant of maize, ZEN. Nevertheless, this was caused by matrix effects rather than low extraction recovery, and can thus be compensated by the use of matrix-matched standards. The method performance characteristics for some analytes are given in the electronic supplementary material. The method has been continuously extended to include 331 bacterial, plant, and fungal metabolites, and has been fully validated for 295 analytes in maize and three other matrices [36]. To successfully acquire as many MRM transitions with acceptable sensitivity and repeatability in a reasonable time, the method was transferred onto the next generation of a QTRAP instrument (QTRAP 5500). As no guidelines are available for multidetection of mycotoxins, the validation procedure was performed according to SANTE 11945/2015 [34], and the trueness of the method was demonstrated with use of samples from organized ring trials. With regard to the apparent recovery in maize, 62% of 295 analytes matched the acceptable recovery range of 70–120% laid down in SANTE 11945/2015 at the highest spiking level. At the levels close to the LOQ, 57% of the analytes fulfilled this criterion. The extent of matrix effects was strongly dependent on the analyte–matrix combinations. No matrix effects were observed for 45% analytes at the highest spiking level and 35% of analytes at the lowest spiking level. The repeatability of the method was acceptable (RSD ≤ 20%) for 95% of the analytes. The trueness of the method was proved by participation in ring trials. The calculated *z* scores were satisfactory for all maize samples analyzed (i.e., between −2 and 2) and also for a broad variety of different matrices, which proves that the method provides accurate results also for other “nonvalidated” matrices.

A critical assessment of extraction methods in the simultaneous analysis of 288 pesticides and 38 mycotoxins was performed in another study [47]. Three extraction procedures were performed for wheat and other matrices: aqueous acetonitrile extraction followed by a modified QuEChERS approach, aqueous acetonitrile extraction, and pure acetonitrile extraction. Different eluent modifiers were used for positive-mode ESI and negative-mode ESI measurements to obtain high sensitivity and sharper peak shape. For positive-mode ESI, two to four times higher responses were observed in the presence of ammonium formate for most of the analytes. Extraction with pure acetonitrile was not efficient in terms of recoveries, whereas the QuEChERS approach and extraction with aqueous methanol showed satisfactory recoveries within the range of 70–120% with RSD less than 20% [34] for most of the analyte–matrix combinations. Although the QuEChERS-like method resulted in lower LOQ and more consistent results, the recoveries were lower in particular for polar analytes [DON 3-glucoside (DON-3-Glc), NIV, T2 tetraol] because of the partitioning step. Finally, QuEChERS-like extraction was chosen as the most suitable approach for the analytes tested [47].

##### SIDA methods

The correction of matrix effects in LC–MS/MS methods using limited sample cleanup can be done with SIDAs; however, this approach also has its limitations. Although the spectrum of commercially available isotopically labeled standards is getting broader, it is still mainly limited to EU-regulated mycotoxins or to those considered by EFSA. Moreover, the cost of these internal standards is far greater than that of those of natural origin, which significantly increases the cost of the analysis [56]. A review of the application of SIDA in mycotoxin analysis was published in 2008 by Rychlik and Asam [61]. Therefore, the following discussion is focused on a few examples of SIDA applications in multimycotoxin LC–MS/MS methods.

A UHPLC–MS/MS method for the determination of all EU-regulated mycotoxins in maize and cereal-based products was developed [70]. The accuracy was enhanced by the application of ^13^C-labeled compounds for each of the target analytes before UHPLC–MS/MS analysis. The simple raw-extract-injection technique was validated as a confirmatory method according to Commission Decision 2002/657/EC [29]. The trueness of the method was verified by the measurement of 12 test materials from different providers with well-defined analyte concentrations.

Method performance parameters were evaluated for maize (see the electronic supplementary material). The sample preparation consisted of two extraction steps: (1) extraction with acetonitrile–water–formic acid (80:19:0.1, v/v/v); (2) extraction of the residue with acetonitrile–water–formic acid (20:79.9:0.1, v/v/v). Both extracts were combined and centrifuged, and the raw extract was fortified with a mixture of labeled standards in a ratio of 4:1 (v/v) before injection. A drawback of this procedure is that more matrix compounds are extracted because of the high water content of the second extraction solvent. However, the use of internal standards efficiently compensated for all matrix effects for all target analytes.

An 11.5-min UHPLC gradient elution using methanol and water containing 5 mM ammonium formate and 0.1% formic acid provided a capacity factor *k*′ of the first analyte eluted (DON) of more than 1 (actually 1.5), and acceptable resolution and peak shape for all analytes, which fulfills the Commission Decision 2002/657/EC criteria [29]. The MS detection was performed using the dynamic MRM mode and fast polarity switching. Dynamic MRM is a technique that monitors the analytes only around the expected retention time, and thus decreases the number of co-occurring MRM transitions, allowing both the cycle time and the dwell time to be optimized for the highest sensitivity, accuracy, and reproducibility. Fast polarity switching needs less than 0.5 s for switching between positive and negative modes, which reduces the losses in sensitivity. As the method fulfilled all the European Commission criteria, it is suitable for routine analysis of maize for official control [33].

A combination of SIDA and SPE cleanup was used in the determination of EU-regulated and EFSA-recommended mycotoxins in cereals. Moreover, conjugated forms of DON (DON-3-Glc, 15ADON, 3ADON) were included [50]. Isotopically labeled standards of 3ADON, T2, enniatins, and beauvericin were prepared in-house. For the ^13^C-labeled equivalents of 15ADON and DON-3-Glc, ^13^C-labeled 3ADON and ^13^C-labeled DON, respectively, were used for correction. Similarly to the previous study [70], internal standards were added to the raw extract, which then underwent SPE cleanup using Bond Elut Mycotoxin cartridges (Agilent Technologies). Special attention had to be paid to the chromatographic separation of DON and DON-3-Glc because of in-source fragmentation of DON-3-Glc. To avoid decreases in sensitivity, the analysis was performed in two single chromatographic runs (positive-mode ESI and negative-mode ESI). Detailed information about the method performance characteristics obtained with use of SIDA is given in the electronic supplementary material. The mycotoxins for which ^13^C-labeled standards were not available were evaluated by matrix-matched calibration of potato starch. The accuracy was confirmed by analysis of commercially available reference materials and samples from interlaboratory testing. This method was later applied to beer [71]. Although satisfactory recoveries were achieved, the LOQ of 20 μg/L for DON-3-Glc is too high for beer control, where common levels of DON-3-Glc are usually lower than 20 μg/L. To achieve a lower LOD and LOQ, the method needs to be optimized further (i.e., use of any cleanup, change of the chromatographic gradient, or use of a more sensitive LC–MS/MS instrument).

A dilute and shoot method for the LC–MS/MS determination of multiple mycotoxins (AFs, OTA, FBs, ZEN, DON, T2, and HT2) in wines and beers has been developed and validated. Separation was accomplished by UHPLC with an analysis time of less than 10 min. Mycotoxins were detected by dynamic MRM in positive-mode ESI. To reduce matrix effects, ^13^C-labeled mycotoxin standards were added to the sample extracts before LC–MS/MS analysis. With external calibration, the recoveries were 18−148% for white wines, 15−118% for red wines, and 20−125% for beers, at three spiking levels. The ^13^C-labeled internal standards compensated for matrix effects effectively, with overall recoveries of 94−112% for white wines, 80−137% for red wines, and 61−131% for beers, with greater recoveries for FBs, at three spiking levels. The RSD was less than 20% for all analytes in the wines and beers. This method was applied in a survey of domestic and imported wines and beers for the determination of OTA, and was extended to include other mycotoxins [72].

## LC–MS for the determination of masked mycotoxins

Masked mycotoxins (also conjugated by plants), which are plant metabolites of mycotoxins, are a well-known and significant subgroup of these natural contaminants. Research on these derivatives has expanded tremendously, especially within the last 10 years, during which time their forms, occurrence, and principles of origin and metabolization in plants have been continuously elucidated. As a result, the development of analytical methods to measure these metabolites has progressed. The topic of masked mycotoxins was comprehensively reviewed by Berthiller et al. [73].

First, the terminology used in the field of masked mycotoxins should be clarified. The term “masked mycotoxins” refers to a specific group of mycotoxin metabolites that are created by plants as their defense against various xenobiotics. The masked mycotoxins can be further subdivided into the two categories of conjugated and bound mycotoxins. Conjugated mycotoxins can be extracted from samples (plants, cereals, food) and detected by further described analytical strategies. In contrast, bound mycotoxins cannot be extracted directly from samples of interest since they are covalently or noncovalently attached to polymeric carbohydrate or protein structures and have to be released from the matrix by chemical or enzymatic treatment first [74].

Regarding analytical methods, basically there are two possible strategies for testing, direct and indirect, depending on the particular analytes and the available laboratory equipment. In the field of direct methods, which cover the soluble forms of these metabolites, the LC–MS approaches are the techniques of choice for accurate, fast, and specific analysis of masked mycotoxins. Unfortunately, for most of the masked mycotoxins analytical standards are not commercially available, and thus in-house purified standards have to be prepared first. This production is laborious and time-consuming.

Since masked mycotoxins tend to be more polar than their parent toxins (glycosylated, sulfated, acetylated forms), their determination can be done easily by an analytical procedure developed for the determination of native forms of mycotoxins. This means, as also previously demonstrated by several authors, that the best recovery of masked mycotoxins can be obtained through extraction with acidified acetonitrile–methanol and water mixtures, which is the procedure widely applied in multimycotoxin analysis. From the available published methods and protocols, it is clear that acetonitrile–water mixtures ranging from 80% to 84% acetonitrile, simple or acidified with 1% acetic acid, are the most versatile extraction solvents, yielding sufficient recoveries (mostly above 70% regardless of the type of matrix) of various masked trichothecenes (DON-3-Glc, T2 glucoside, HT2 glucoside, NIV glucoside) or ZENs (ZEN 14-glucoside, ZEN 14-sulfate). Although several studies dealt with various cleanup strategies, it was uniquely determined that neither SPE cartridges nor IACs are suitable for their analysis, since only very low recoveries (less than 50%) are commonly obtained [73].

Generally, the dilute and shoot strategy has been proven to provide the best recoveries and data repeatability. In contrast, the QuEChERS approach provides significantly lower recoveries of masked forms compared with the parent compounds. In the case of the QuEChERS approach, analytes are typically not transferred into the acetonitrile phase because of their polar character, which also causes retention problems in SPE cartridges. Chromatographic separation can be easily achieved by use of traditional C_18_ columns, although methods based on hydrophobic interaction LC could also be advantageous.

Problems as a result of the lack of analytical standards can be avoided by the application of indirect methods when primary hydrolysis (enzymatic, acetic, and basic) of conjugated mycotoxins is required before analysis [73]. The significant disadvantage of these methods is the impossibility of complete hydrolysis of conjugates and its confirmation, which results in problematic quantification and underestimation of results.

## High-resolution (LC–HRMS) approaches for the targeted determination of mycotoxins

Currently, the use of HRMS is gaining increasing interest in both research and routine laboratories. Advanced HRMS instruments combine crucial features such as increased selectivity and mass resolution, lower cost, and relatively easy maintenance. HRMS is provided by two types of analyzers, TOF and Orbitrap analyzers, with resolving power of 10,000–100,000 and 140,000–240,000 (full width at half maximum defined at *m*/*z*), respectively. In contrast to MS/MS, HRMS techniques can overcome all the limiting factors of SIM/MRM analyte detection, thus representing an appropriate alternative to the use of QqQ instruments for targeted as well as nontargeted compound detection. Generally, HRMS measurements have enhanced performance in terms of confirmatory capabilities compared with MS/MS measurements. Especially when one is dealing with complex sample matrices, adequate mass resolution is essential, and the absence of noise can cause a significant decrease of the signal-to-noise ratio [47]. The use of accurate mass measurement permits full spectral data acquisition for all ions, does not rely on fragmentation of analytes, which means that it can overcome the problems caused by production of transitions (nonspecific transitions and stable adducts), and allows retrospective data mining of the chromatogram to look for additional compounds of interest, predominantly metabolites. Overall, HRMS analyzers operate in full-scan mode, and thus allow target, posttarget, and nontarget analysis in a single run without time-consuming optimization of MRM conditions for each compound. The analysis of accurate mass reduces matrix interferences within one mass unit of the target analytes that are normally detected in QqQ systems; however, sole measurement of accurate mass for analyte identification can lead to false positive results or misidentifications.

A new generation of hybrid instruments, quadrupole–TOF (QTOF) and Q–orbital ion trap (Q Exactive) instruments, combine the benefits of high-performance quadrupole selection of precursor ions with those of high-resolution mass detection. Furthermore, the use of these systems also allows higher selectivity in comparison with targeted QqQ systems and simultaneously allows retroactive processing of samples for other untargeted compounds of interest. To collect MS/MS data for nontargeted compounds either a data-dependent acquisition (DDA) strategy or a data-independent acquisition (DIA) strategy can be used. In DDA, a limited number of ions with highest abundance detected in the full MS scan are isolated and fragmented in a product ion scan experiment. The approach based on DIA involves sequential isolation of windows across a mass range for MS/MS. The cycle is repeated throughout the LC run ensuring that product ion spectra of all ions are recorded. Moreover, the DIA approach allows the use of fragment ions for quantification [75]. Application of DIA and DDA in analysis of mycotoxins was demonstrated in several recent studies [76, 77].

Finally with respect to Commission Decision 2002/657/EC [29] for HRMS measurements, each ion earns two identification points, so quantification with the molecular ion and confirmation with one product ion gives enough points to confirm any substance. The advantages and disadvantages of LC–HRMS approaches over targeted LC–MS/MS are summarized in Table 3.

**Table 3:** Comparison, advantages and drawbacks of mass spectrometry (MS) instrumentation

MS technique	Analyzer	Pros	Cons	
LRMS(/MS)	QqQ, IT, QLIT	High sensitivity and selectivity in MRM mode Wide linear dynamic range Robust gold standard instrumentation Lower purchase costs	Number of simultaneously detected analytes in MRM mode is limited Poor/moderate sensitivity in full MS mode Need to optimize detection conditions for each analyte Screening without reference standard not usually possible	
HRMS(/MS)	TOF, QTOF	High sensitivity and selectivity in full MS mode Postacquisition data interrogation for nontargeted analytes Identification of unknowns is possible	Narrower dynamic range compared with LC–MS(MS/MS) instruments High purchase cost	Lower mass resolving power and mass accuracy compared with Orbitrap systems
	Orbitrap, Q–Orbitrap			Acquisition speed limited at high mass resolving power settings

*HRMS* high resolution mass spectrometry, *LC* liquid chromatography, *LRMS* low-resolution mass spectrometry, *MRM* multiple-reaction monitoring, *QLIT* quadrupole–linear ion trap, *Q–Orbitrap* quadrupole–Orbitrap, *QqQ* triple quadrupole, *TOF* time of flight

Despite the ability of HRMS instruments to simultaneously detect various analytes that can be confirmed by MS/MS, this technique is not extensively used for multimycotoxin analysis, and only a couple of methods have been published so far (Fig. 3). Tanaka et al. [78] successfully applied the combination of LC–atmospheric pressure chemical ionization TOF MS for the determination of trichothecenes, ZEN, and AFs in cereal-based food. However, additional SPE cleanup of samples was needed to obtain sufficient sensitivity for all analytes. This pioneering study was followed by several others, and a comprehensive study by Mol et al. [65]. In that study, four different extraction approaches and UHPLC–TOF MS and MS/MS for the determination of mycotoxins, pesticides, plant toxins, and veterinary drugs were compared. It was shown that a procedure using water–acetonitrile–1% formic acid is applicable for the extraction of multiple food contaminants from different matrices. UHPLC–TOF and UHPLC–Orbitrap mass analyzers were applied to examine major *Fusarium* mycotoxins (FBs, DON, 3ADON, 15ADON, NIV, HT2, T2, ZEN, DON-3-Glc, FUS-X) in cereals. QuEChERS and aqueous acetonitrile extractions were applied before instrumental determination of the analytes. From published results, it can be concluded that both techniques are fit for purpose for the determination of the mycotoxins tested, but the approach using TOF MS requires an additional cleanup strategy to achieve sufficient sensitivity for all targeted analytes [79]. A hybrid QTOF system for the determination of trichothecenes in wheat, corn, rice, and noodles was used in another study [80].

The most comprehensive studies describing the use of HRMS techniques in multimycotoxin analysis were published by Herebian et al. [81], Rubert et al. [52], De Dominics et al. [82], and Beccari et al. [53]. These articles cover the main mycotoxin representatives of *Fusarium*, *Claviceps*, *Aspergillus*, *Penicillium,* and *Alternaria* fungi and the applicability of TOF and Orbitrap MS systems for both screening and quantitative analysis of the respective toxins in cereal-based matrices. In one study [81] the determination of undiluted acetonitrile–water extracts of cereals was with HPLC–MS/MS and microcapillary HPLC–LTQ Orbitrap MS instruments. It was also concluded that the HRMS technique is fully usable and a time-saving method for rapid and accurate analysis of mycotoxins. LC–Orbitrap MS instrumentation was used for the determination of all legislatively regulated mycotoxins in wheat and barley flours and crisp bread [52]. With the support of the SPE cleanup of extracts, it was possible to quantify all desired mycotoxins, and all validation data met the current European regulatory requirements for LC–MS confirmatory analysis [29]. Critical evaluation of four different extraction procedures (modified QuEChERS extraction, matrix solid-phase dispersion, solid–liquid extraction, and SPE cleanup) for simultaneous determination of 32 different mycotoxins was conducted [52]. Separation and detection of the analytes was performed by the UHPLC–Orbitrap MS method. The only extraction procedure that was mutually developed with the HRMS method and capable of determining all mycotoxins tested was the QuEChERS procedure (see Table 2).

## LC–HRMS-based approaches for the qualitative screening of mycotoxins

LC–HRMS-based techniques have clearly been shown to be applicable as reliable detection tools for the screening of conjugated mycotoxins and various mycotoxin metabolites for which no analytical standards are available. The increasing interest in the use of HRMS is evident from the number of articles that have been published [51, 83, 84].

The presence of glycosylated metabolites of HT2 and T2 in wheat, oat, and barley was confirmed by Veprikova et al. [83]. Cereal extracts were purified through IACs, which allowed the purification and preconcentration of the glycosylated metabolites. The use of this specific cleanup in combination with a UHPLC–QqTOF system operating in MS and MS/MS modes allowed the identification of HT2 glucoside and T2 glucoside. Moreover, HT2 diglucoside and T2 diglucoside in barley were found for the first time [83]. The occurrence of HT2, T2, and their glycosides in cereals and their fate during malting were studied [51]. Because of the use of an HPLC–Orbitrap system (HRMS), a retrospective analysis of the full-scan HRMS chromatograms was possible, and presence of neosolaniol, DAS, and their monoglucosides was also successfully evaluated. The study confirmed a common co-occurrence of HT2, T2, HT2 glucoside, and T2 glucoside in wheat, oat, and barley raw grains. Furthermore, also traces of neosolaniol glucoside and DAS glucoside were found. The preliminary investigation on the fate of HT2 and T2 and their glucosylated forms during malting revealed a general mycotoxin reduction from cleaned barley to malt [51].

In another study, novel masked mycotoxins, FUS-X glucoside and NIV glucoside, were identified in wheat with use of an HPLC–Orbitrap system operating in full-scan and in-source MS/MS modes. The extract was purified by an SPE column to achieve higher signal for these analytes and thus facilitate their reliable identification. Both glucosides were detected in negative mode with a mass deviation of less than -1.1 ppm for [M − H]^-^ and -0.83 ppm for [M − CH_3_COO]^-^ [84].

## Metabolomics approaches to study mycotoxin metabolism in cereals

Recently, metabolomics-based approaches have found their place in the mycotoxin research field to (1) study the metabolism of mycotoxins in grains, especially in resistant plant varieties such as *Fusarium*-resistant wheat [85], (2) explore the co-occurrence of these secondary fungal metabolites with plant metabolites, and (3) reveal the formation of so far unknown metabolites that can also be present in cereal-based food. In line with this, two different strategies can be distinguished: targeted and untargeted metabolomics. In targeted approaches, the abundances of metabolites of a set of predefined known substances are determined. Such an approach allows absolute quantification but it is usually limited to metabolites for which reference standards are available. Untargeted approaches aim to record MS features of all detectable compounds, including those unknown at the time of sample analysis. This approach has therefore the advantage of probing the entire metabolic space and can obtain relative abundances of several hundred known and unknown metabolites. Both targeted and untargeted LC–HRMS-based metabolomic studies follow a general workflow consisting of several steps: (1) experimental design, (2) sample preparation, (3) chromatographic separation and MS detection, (4) data acquisition, (5) data processing, and (6) data analysis and interpretation. These workflows were recently summarized in several comprehensive reviews [86–88]. Several studies dealing with the biotransformation of mycotoxins in plants using a recently developed untargeted stable isotope labeling (SIL)-assisted approach using ^13^C-labeled standards in combination with LC–HRMS have been published [89–91]. All the studies share a common experimental setup. Briefly, the plant ears were spiked with a 1:1 mixture of ^12^C toxin and ^13^C-labeled toxin in triplicate at different time points depending on the experiment. As blank control samples, “mock” (i.e., water) treated plant ears were prepared. The harvested ears frozen at -80 °C were milled, and then extracted with a common extraction mixture used for mycotoxins. A UHPLC–LTQ Orbitrap XL system equipped with an ESI source was used. The chromatography columns (commonly C_18_) and gradient as well as the HRMS conditions were chosen and set on the basis of the parent toxin studied.

Qualitative analysis of LC–HRMS data was performed with Xcalibur 2.1.0 QualBrowser, and for relative and absolute quantification of known analytes Xcalibur 2.1.0 QuanBrowser was used. Data were further evaluated with MetExtract [92], which was programmed to automatically extract the corresponding MS peak pairs in mass spectra of a 1:1 mixture of the sample containing^12^C toxin and ^13^C-labeled toxin. The metabolites were putatively identified on the basis of the specific criteria [92].

**Fig. 4:**
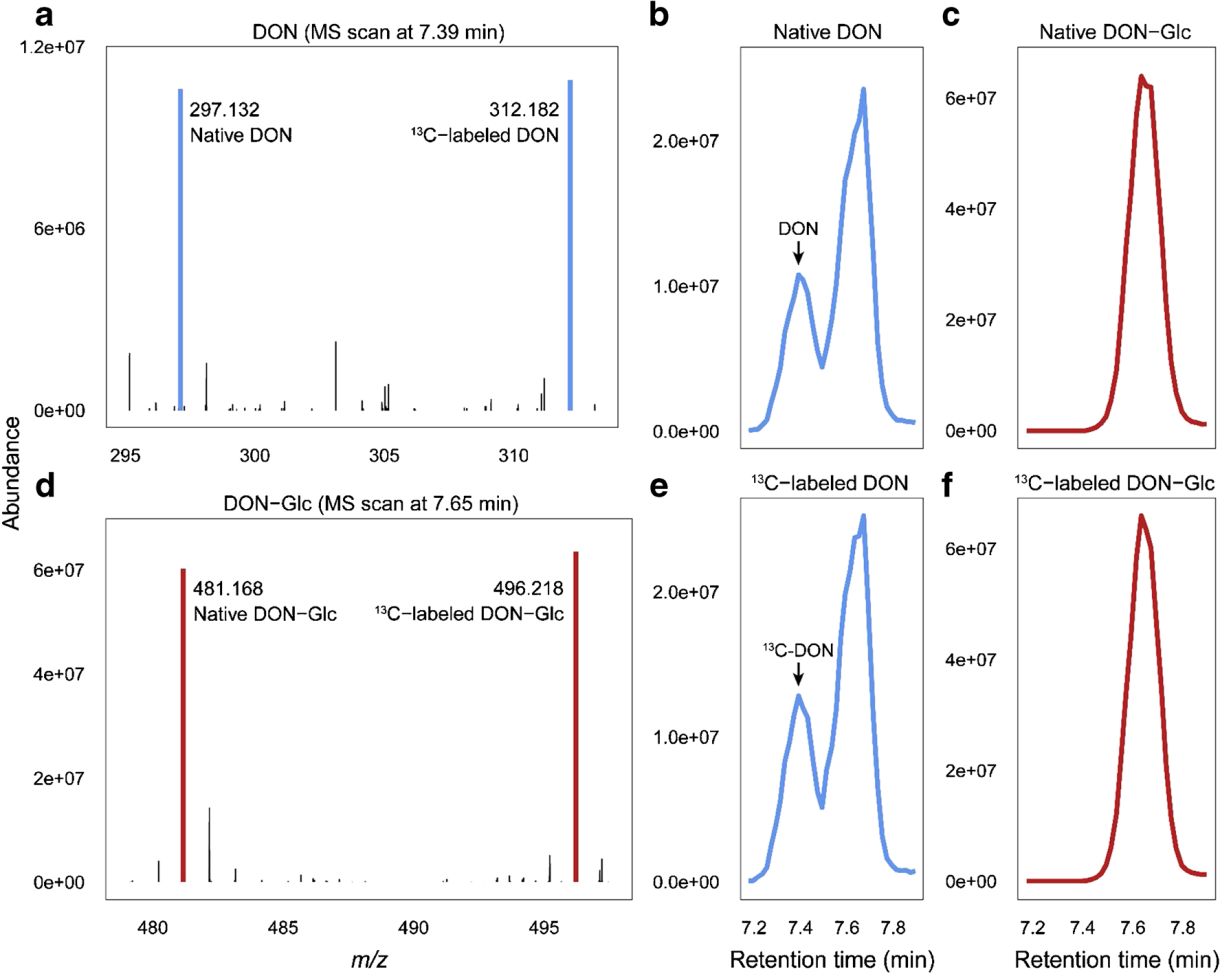
Metabolism of deoxynivalenol (DON) in wheat. Sample treated with 1:1 native DON and ^13^C-labeled DON (tracer). Deoxynivalenol 3-glucoside (DON-Glc) was found as a major metabolite. Mass spectrometry (MS) scan of DON and ^13^C-labeled DON (A). Extracted ion chromatogram of DON (B). Extracted ion chromatogram of DON-Glc (C). MS scan of DON-3-Glc and ^13^C-labeled DON-Glc (D). Extracted ion chromatogram of ^13^C-labeled DON (E). Extracted ion chromatogram of ^13^C-labeled DON-Glc (F)

In a DON-metabolism study in wheat, MetExtract data processing revealed a total of 57 ion pairs in the full-scan chromatogram of DON-treated samples that passed the aforementioned criteria. DON and its nine biotransformation products were detected in wheat samples treated with a 1:1 mixture of ^12^C DON and ^13^C-labeled DON (Fig. 4). All DON metabolites identified contained the intact carbon skeleton of DON in their molecular structure. Moreover, no DON biotransformation products containing fewer than 15 carbon atoms were detected. DON-3-Glc was found as a main metabolite, which was confirmed by measurement of an analytical standard. Beside glycosylation, the glutathione pathway, where DON *S*-cysteine and DON *S*-cysteinylglycine were identified, was described in wheat for the first time in this study [89]. Moreover, five unknown DON conjugates were found.

Combination of SIL and LC–Orbitrap MS in fast polarity switching mode followed by MetExtract data processing was applied in a study of HT2 and T2 metabolism in barley. Additionally, a QTOF instrument was used for acquisition of MS/MS spectra, which were needed for further structure annotation [90]. In total, nine HT2 and 13 T2 metabolites were annotated and partly identified. The metabolism routes in barley covered hydrolysis of acetyl and isovaleryl groups, and hydroxylation as well as covalent binding of glucose, malonic acid, acetic acid, and ferulic acid. A major metabolite of HT2 and T2 metabolism, HT2 3-*O*-β-glucoside, formed at the maximum level as soon as the first day after toxin application and was further metabolized. Other putatively identified metabolites included 15-acetyl-T2 tetraol malonylglucoside, hydroxy-HT2 glucoside, hydroxy-HT2 malonylglucoside, HT2 diglucoside, HT2 malonylglucoside, and feruloyl-T2 [90].

Similarly, a QTOF system in MS and MS/MS mode was used for the identification of HT2 and T2 metabolites in wheat [91]. Together with the use of SIL, it allowed putative annotation of 11 HT2 and 12 T2 metabolites. It was confirmed that the metabolism route did not differ from that in barley (i.e., T2 was rapidly converted to HT2, which was further metabolized to HT2 3-*O*-β-glucoside). In contrast to DON metabolism in wheat, no glutathione metabolite was found in case of HT2 and T2.

Untargeted SIL profiling using sensitive HRMS instrumentation is undoubtedly a highly efficient tool for studying mycotoxin metabolism in plants. Currently, this technique is being used during baking on an industrial scale to reveal the formation of degradation products of mycotoxins. Moreover, such tracer-fade studies could also be used for elucidation of elevated levels of masked mycotoxins found during malting and brewing [93, 94].

## Conclusions and outlook

One of the biggest challenges in mycotoxin analysis is still the sampling issue, for which guidance has become available [33]. However, it remains a difficult and tedious task to obtain a representative sample. Subsequent extraction with appropriate solvents that match the range of multiple co-occurring mycotoxins to be determined is another crucial step, followed by proper cleanup. The latter is dependent on the final determination step, ideally involving chromatography and MS. Chromatography will continue to play a crucial role in the determination of mycotoxins unless a radically different approach to separate complex mixtures is developed. With the advent of small particles in relation to UHPLC, smaller amounts of samples can be processed faster than ever. To quantify the wealth of potential mycotoxins and other potentially toxic substances in our food and feed chain in highly complex matrices, separation remains as important as ever. The great increases in sensitivity and selectivity of LC–MS instruments have made a significant contribution in qualitative and quantitative determination of mycotoxins in cereal-based food and other commodities. However, matrix effects, isobaric interference, and maintaining confidence in the assignment of identity are still the major limitations for LC–MS methods used for the quantification and identification of food contaminants, including mycotoxins, in complex matrices. The increasing use of HRMS instruments has reduced the problems associated with low selectivity and errors in identification because of the capability of accurate mass measurement. However, how to fully eliminate matrix effects has not been fully technically solved yet.

Despite these remaining challenges, dilute and shoot approaches before LC–MS/MS, which do not require any cleanup, are increasingly being used for the quantification of several hundred mycotoxins and other secondary metabolites of fungi and plants in food and feed matrices. Moreover, for all regulated toxins, matrix effects and especially signal suppression can be compensated for through the use of fully ^13^C-labeled internal standards, which have become commercially available. Ensuring comparability of measurement results is another challenge especially for mycotoxin–commodity combinations for which no certified reference materials exist.

In the past few years, mycotoxin analysis has been moving from the targeted analysis of individual mycotoxins to untargeted metabolite profiling and metabolomics of (ideally) all secondary metabolites that are involved in plant–fungi interactions. The major method used in this area is based on in vivo stable isotope ^13^C labeling and subsequent measurement of biological samples by full-scan high-resolution LC–MS. We anticipate SIL will become a major technique to study the fate of mycotoxins during food processing. SIL can also be used to detect deviations of secondary metabolites of fungi, plants, and bacteria from normal patterns, flagging suspicious food and cereal samples for further analysis and confirmation, and for more accurate quantification and identification of compounds.

## Electronic supplementary material

ESM 1 (PDF 4.86 kb)

ESM 2 (XLSX 12.8 kb)
