# Supplementary material for: Advanced LC–MS-based methods to study the co-occurrence and metabolization of multiple mycotoxins in cereals and cereal-based food
Source: Anal Bioanal Chem. 2017 Dec 22;410(3):801–25. doi: 10.1007/s00216-017-0750-7 (PMC5775372; doi:10.1007/s00216-017-0750-7)
Supplement: Supplementary file 1 — (PDF 4.86 kb) [file 216_2017_750_MOESM1_ESM.pdf]

Analytical and Bioanalytical Chemistry

Electronic Supplementary Material

Advanced LC–MS-based methods to study the co-occurrence and metabolization of multiple mycotoxins in cereals and cereal-based food

Alexandra Malachová, Milena Stránská, Marta Václavíková, Christopher T. Elliott, Connor Black, Julie Meneely, Jana Hajšlová, Chibundu N. Ezekiel, Rainer Schuhmacher, Rudolf Krska
